# Exploring the links between population density, lifestyle, and being overweight: secondary data analyses of middle-aged and older Chinese adults

**DOI:** 10.1186/s12955-019-1172-3

**Published:** 2019-06-11

**Authors:** Ruoyu Wang, Zhixin Feng, Desheng Xue, Ye Liu, Rong Wu

**Affiliations:** 1 School of Geography and Planning, Sun Yat-Sen University, Xingang Xi Road, Guangzhou, 510275 China; 2 Guangdong Key Laboratory for Urbanization and Geo-simulation, Sun Yat-Sen University, Xingang Xi Road, Guangzhou, 510275 China; 3 Primary Care and Population Sciences, Faculty of Medicine, University of Southampton, Southampton, UK

**Keywords:** Population density, Sedentary lifestyle, Overweight, Neighbourhood, China

## Abstract

**Background:**

The increasing prevalence of obesity across all age groups has become a major health concern in China. Previous studies have found strong links between population density, sedentary lifestyle, and the risk of being overweight among adults and adolescents in Western countries. However, little research has been conducted to disentangle this relationship in China, which is rapidly urbanizing and densely populated. Compared to other age groups, middle-aged and older adults tend to have a higher risk of being overweight, which increases their risk of diabetes, high blood pressure, and other weight-related chronic diseases. In addition, they are especially sensitive to neighbourhood environmental factors such as population density. Therefore, we aimed to unravel the link between population density and the risk of being overweight among Chinese middle-aged and older adults, with a particular focus on the mediating role of lifestyle choices.

**Methods:**

Data from the 2011 China Health and Retirement Longitudinal Study were analysed. Individuals (*N* = 5285) were sampled from 405 neighbourhoods nested within 150 cities. Body Mass Index (BMI) was calculated based on self-reported body weight and height (being overweight was defined as a BMI ≥ 24 kg/m^2^). Multilevel regression and mediation analyses were applied to explore associations between population density, a sedentary lifestyle, and the risk of being overweight.

**Results:**

Middle-aged and older adults who lived in densely populated neighbourhoods had higher odds of being overweight. Further, this link was mediated by residents’ mode of travel and physical exercise; specifically, these residents had higher odds of owning a car and spending lesser time on weekly physical exercise, thereby increasing their risk of being overweight. Furthermore, the association between car ownership and the odds of being overweight varied by neighbourhood population density.

**Conclusions:**

There was a positive association between neighbourhood population density and middle-aged and older adults’ risk of being overweight. This relationship may exist because people who live in densely populated neighbourhoods tend to lead a sedentary lifestyle. Our findings also suggest that, in rapidly urbanizing countries, a sedentary lifestyle may be especially harmful to middle-aged and older adults who live in densely populated neighbourhoods.

## Background

In China, the proportion of overweight adults increased from 22.8 to 30.1% between 2002 and 2012 [1]. Being overweight is associated with various diseases, including cardiovascular disease [2, 3], diabetes [2, 3], and cancer [4, 5], and it increases individuals’ mortality risk [4]. On the other hand, being overweight is related to psychological problems, such as low self-esteem [6]; it can result in negative emotions that may lead to chronic stress in their daily life or even suicide [7]. All these could affect individuals’ Health-related quality of life (HRQoL) which is defined as people’s subjective evaluation on different aspects of health status [8].

China has experienced a rapid process of urbanization over the last 40 years. The percentage of the total population that lived in cities was only 18% in 1978, and unprecedentedly increased to more than 56% in 2015 [9]. In addition, more than one billion people are expected to live in urban areas by 2030 [10]. Previous studies have shown that population density may be a double-edged sword to residents’ health in developing countries. On the one hand, high population density is normally associated with poor sanitation, shortage in public facilities and services, and environmental degradation [11]. On the other hand, high population density is conducive to the development of healthy lifestyles such as more physical activities and lower rates of car use, which could reduce the risk of being overweight [12–15]. Identifying the relationship between population density and being overweight may be necessary to improve people’s quality of life and an urgent public health issue in China.

Regarding mechanisms through which population density affects health, a significant body of research in developed countries has noted the importance of lifestyle as a mediating component of health protection [16–25]. Indeed, the healthy lifestyle theory states that lifestyle is a bridge between environmental and human factors that affects health outcomes [16]. Previous studies have concluded that there are two main lifestyle choices through which population density reduces the risk of being overweight in developed countries. First, ‘Transportation mode’ [14, 17–25] and the ‘3D’ theory (population Density, pedestrian-friendly Design, and a diversity of Destinations) claim that population density may increase the walkability of a neighbourhood [20, 25] and reduce the distance to amenities [14, 17, 18]. Therefore, residents living in communities with a higher population density would prefer walking and using public transport to private transport, thereby increasing their engagement in physical exercise, which invariably reduces the risk of being overweight [21, 24]. Second, a neighbourhood with high population density may also increase the time that residents spend on physical exercise [12, 13, 21, 24]. For instance, a high population density is closely related to a perceived level of safety (i.e. fewer crimes and gangs) and a heightened sense of safety [12, 13]. Therefore, middle-aged and older adults are more likely to engage in outdoor activities (including outdoor exercises) when their neighbourhood is perceived to be safe [12]. Thus, engaging in outdoor activities can potentially reduce their risk of being overweight. In addition, high population density may encourage people to connect with their neighbours, thereby increasing the level of neighbourhood social cohesion [12, 21]. Compared to younger residents, middle-aged and older residents are more afraid of being injured when exercising alone. Therefore, older residents are more willing to exercise in a cohesive neighbourhood, where people are likely to help each other in the event of an injury [12].

Although existing studies have found that living in a densely populated neighbourhood is associated with a decreased risk of being overweight in developed countries [16–25], only a few studies have attempted to unravel the relationship between population density and the risk of being overweight in China [26–39]. Further, these studies have focused only on a single city or region, thereby limiting the generalizability of the findings. Moreover, none of these studies has explored the mediating effect of lifestyle choices on the relationship between neighbourhood population density and the risk of being overweight.

**Fig. 1:**
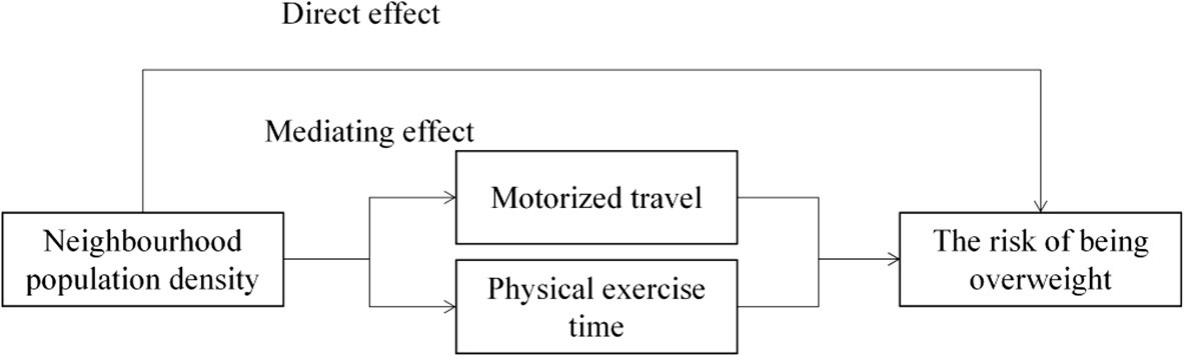
Framework of the relationship between neighbourhood population density, lifestyle, and being overweight

To bridge these gaps in literature, we investigated the relationship between neighbourhood population density, individuals’ lifestyle, and the risk of being overweight in China, using data from the 2011 wave of China Health and Retirement Longitudinal Study (CHARLS). We particularly focused on the mediating effect of lifestyle choices on the relationship between population density and the risk of being overweight. This study contributes to the body of existing literature in two respects: first, it improves our understanding of how neighbourhood population density and a sedentary lifestyle influence middle-aged and older adults’ risk of being overweight in China; second, it offers a deeper understanding of the social and behavioural pathways that link urbanisation to the prevalence of being overweight in the Chinese context. The conceptual framework of the current research is shown in Fig. 1. We hypothesized that neighbourhood population density would directly affect residents’ risk of being overweight and indirectly affect the likelihood of being overweight through the prevalence of a sedentary lifestyle.

## Methods

### Data

We used data from the 2011 wave of CHARLS, which was conducted by the National Development Research Institute of Peking University in cooperation with the China Social Science Research Centre and the Youth League Committee of Peking University [30]. The study used a cross-sectional design wherein data was collected between the months of April and August 2011. Respondents were selected using the probability proportional to size (PPS) sampling technique in which the probability of selecting a sampling unit is directly proportional to its population size. The advantage of PPS is that it can generate representative sampling units without much cost and that the usage of auxiliary information can reduce sampling error. In the first stage of sampling, 150 prefecture-level divisions from 30 provinces were randomly selected depending on the population size of each prefecture-level division. In the second stage, 450 neighbourhoods were randomly selected from the sampled prefecture-level divisions, depending on the population size of each neighbourhood. The neighbourhood (*she qu*) that served as the basic sampling unit for CHARLS refers to the fifth-level of the basic administrative division with defined boundaries and elected heads. The data set included 17,587 individuals; however, a key question about ‘weekly physical exercise time’ was asked to a randomly chosen sub-sample of households. Therefore, the final data set included 5285 individuals nested within 405 neighbourhoods.

### Variables

#### Outcome

Weight and height were measured in the CHARLS. BMI was calculated as the ratio of body weight to height squared (kg/m^2^). According to the ‘Nutrition improvement work management method’, enacted by the National Health and Family Planning Commission of the People’s Republic of China, being overweight was defined as a BMI ≥ 24 [31].

#### Predictor

##### Population density at the neighbourhood level

The number of residents per square kilometre (km^2^) at the neighbourhood level was used to measure neighbourhood population density. This predictor is a reflection of population agglomeration in the neighbourhood, which is one of the most widely used proxies of urbanity [26].

##### Lifestyle indicators

In this study, we considered sedentary lifestyle as a mediator, since it is directly related to calorie metabolism. Two kinds of sedentary lifestyle indicators were examined: residents’ transportation mode and weekly physical exercise time. People whose family owns a car may prefer driving instead of walking, which means that they may burn fewer calories during their daily commute. Therefore, we defined transportation mode based on whether or not their family owned a car (1 = with car ownership; 0 = without car ownership). Weekly physical exercise (including taking a walk) was self-reported by respondents; the number of hours spent on physical exercise was used as the unit of analysis.

#### Covariates

We controlled for a series of individual-level covariates including sex, age, marital status, education, and annual household income per capita [23, 27]. In addition, previous studies have indicated that people’s health-related habits and resources may also increase their risk of being overweight [14, 26]. Therefore, variables such as eating frequency (i.e. 1 if the respondent consumes meals more than three times a day; 0 if the respondent consumes meals no more than three times a day), smoking history, drinking history, medical insurance status, and physical health status (i.e. respondents who reported having any chronic disease were assigned a value of 1), were also controlled. Neighbourhood social and built environment have also been found to affect the likelihood of being overweight [14, 26]. Therefore, annual neighbourhood income per capita, neighbourhood sports facilities, and neighbourhood sports groups were also controlled. In China, there is a wide disparity between urban and rural areas in terms of lifestyle, provision of health-related facilities, and quality of life; such a disparity may pose different risks to being overweight. Therefore, urban-rural residents were also controlled. For continuous variables such as household income per household member and neighbourhood income per neighbourhood resident, we applied their logarithmic form, since the original form was not normally distributed and was affected by the existence of outliers. Summary statistics for the study variables are presented in Table 1. Specifically, we have reported the proportion of respondents across the categories of the binary variable and across the three quartiles for continuous variables.

**Table 1:** Descriptive statistics for the study variables

Variable	Total sample	Overweight sample	None-overweight sample
	Proportion/median (P25-P75)	Proportion/median (P25-P75)	Proportion/median (P25-P75)
Dependent variable			
BMI status (ref: BMI < 24 kg/m^2^)			
Overweight (BMI ≥ 24 kg/m^2^)	0.41		
Independent variables			
Motorized traveller (ref: No)			
Yes	0.09	0.16	0.04
Time spent on weekly physical exercise (hours)	28.50 (14.00–38.00)	21.00 (3.00–29.00)	28.00 (14.00–46.00)
Population density (person/km^2^)	725.62 (257.31–71,392.85)	897.88 (379.74–95,000.00)	574.99 (221.02–15,000.00)
Control variables (demographic and socioeconomic variables)			
Sex (ref: Female)			
Male	0.45	0.39	0.49
Age (years)	58.00 (51.00–68.00)	57.00 (49.00–62.00)	58.00 (52.00–69.00)
Marital status (ref: Single, divorced, or widowed)			
Married	0.88	0.91	0.86
Education (ref: Primary school or below)			
Junior high school	0.20	0.23	0.19
Senior high school or above	0.11	0.14	0.08
Living area (ref: Rural area)			
Urban area	0.38	0.46	0.32
Eating frequency (ref: ≤ three times a day)			
> three times a day	0.87	0.89	0.86
Smoking history (ref: Non-smoker)			
Smoker	0.38	0.31	0.42
Drinking history (ref: Non-drinker)			
Drinking	0.32	0.29	0.35
Medical insurance status (ref: No)			
Yes	0.93	0.93	0.94
Physical health status (ref: Do not have a disease)			
Have a disease	0.65	0.71	0.61
Annual household income per capita (Chinese Yuan)	4794.00 (1210.00–57,926.39)	4340.00 (1400–60,500.00)	3410.00 (1040.00–20,200)
Annual neighbourhood income per capita (Chinese Yuan)	3018.00 (1423.00–8800.00)	3018.00 (1500.00–12,000)	3000.00 (1400.00–20,000.00)
Neighbourhood sports facilities (ref: No)			
Yes	0.64	0.65	0.64
Neighbourhood sports groups (ref: No)			
Yes	0.52	0.55	0.50

### Data analysis

Due to the hierarchical structure of the data (individuals nested within neighbourhoods), multilevel models were deemed suitable for this research. We examined the effects of population density and lifestyle on respondents’ risk of being overweight, and whether a sedentary lifestyle mediates this relationship, using a two-level regression analysis.

We used multilevel regression analysis along with mediation analysis for the current study. First, we estimated the association between neighbourhood population density and the risk of being overweight using multilevel logistic regression analysis (Model 1). Second, we estimated the association between neighbourhood population density and the likelihood of owning a car using multilevel logistic regression analysis (Model 2). Subsequently, we estimated the linkage between neighbourhood population density and the amount of time spent on weekly physical exercise using multilevel liner regression analyses (Model 3). Third, following Baron and Kenny’s (1986) mediation analysis approach [32], we regressed the risk of being overweight on the level of neighbourhood population density, while controlling for the mediators (i.e., lifestyle choices); Sobel test and other mediation tests were used to test the significance of the mediation effect (Model 4a). Then, we added cross-level interaction effects to examine whether neighbourhood population density moderated the relationship between lifestyle choices and the risk of being overweight, using multilevel logistic regression analyses (Model 4b). All continuous variables were centred on the grand mean in the interaction. All analyses were conducted using STATA 15.0.

## Results

Table 2 shows the results of the multilevel logistic regression analysis: Model 1 shows that the odds of being overweight increased with higher neighbourhood population density (OR = 1.103, 95% CI = 1.056–1.153). Further, the odds of being overweight decreased with age (OR = 0.972, 95% CI = 0.965–0.979), and married respondents were more likely to be overweight than their single, divorced, and widowed counterparts (OR = 1.231, 95% CI = 1.004–1.509). Respondents with higher educational attainment were also more likely to be overweight than those who were less educated (OR = 1.338, 95% CI = 1.096–1.633). Compared to rural respondents, urban respondents were more likely to be overweight (OR = 1.620, 95% CI = 1.363–1.926). Interestingly, both drinking (OR = 0.753, 95% CI = 0.639–0.889) and smoking (OR = 0.863, 95% CI = 0.748–0.997) were related to a decreased risk of being overweight. Lastly, respondents with a physical disease were more likely to be overweight (OR = 1.747, 95% CI = 1.535–1.988). Model 2 shows the results of the respective multilevel model on respondents’ lifestyle choices, which suggests that the odds of owning a car increases with neighbourhood population density (OR = 1.421, 95% CI = 1.260–1.604). Model 3, on the other hand, shows that the amount of time that respondents devoted to physical exercise decreased with higher neighbourhood population density (B = − 0.066, SE = 0.017).

**Table 2:** Multilevel regression estimates for models with overweight respondents (Model 1), motorized travellers (Model 2), and the Logarithm of time spent on physical exercise (Model 3), as dependent variables

Variable	DV: Overweight	DV: Motorized traveller	DV: Logarithm of time spent on physical exercise
	Model 1	Model 2	Model 3
	OR (95% CI)	OR (95% CI)	B (SE)
Fixed part			
Independent variable			
Logarithm of neighbourhood population density	1.103*** (1.056–1.153)	1.421*** (1.260–1.604)	−0.066*** (0.017)
Individual-level attributes			
Male (ref: female)	0.885 (0.742–1.056)	0.734* (0.532–1.013)	0.142*** (0.042)
Age	0.972*** (0.965–0.979)	0.965*** (0.949–0.982)	−0.024** (0.002)
Married (ref: single, divorced, or widowed)	1.231** (1.004–1.509)	1.403 (0.870–2.262)	0.109** (0.052)
Educational attainment (ref: ≤ primary school)			
Junior high school	1.152* (0.987–1.343)	2.410*** (1.747–3.326)	−0.114*** (0.041)
≥ Senior high school	1.338*** (1.096–1.633)	8.339*** (5.831–11.926)	−0.093 (0.060)
Living in an urban area (ref: rural)	1.620*** (1.363–1.926)	1.509** (1.012–2.250)	−0.225*** (0.073)
Having meals ≤ three times a day (ref: meals > three times a day)	1.093 (0.889–1.345)	1.327 (0.823–2.139)	0.275*** (0.063)
Smoker (ref: non-smoker)	0.753*** (0.639–0.889)	1.195 (0.832–1.717)	0.002 (0.039)
Drinking (ref: non-drinker)	0.863** (0.748–0.997)	1.009 (0.742–1.370)	0.128*** (0.038)
Having medical insurance (ref: no)	0.944 (0.748–1.192)	1.274 (0.773–2.100)	0.206*** (0.070)
Having a physical disease (ref: no)	1.747*** (1.535–1.988)	1.224 (0.937–1.600)	0.017 (0.034)
Logarithm of household income per capita	1.004 (0.979–1.030)	1.062** (1.005–1.121)	0.032*** (0.009)
Neighbourhood-level attributes			
Logarithm of neighbourhood income per capita	0.992 (0.960–1.024)	1.015 (0.938–1.099)	0.023 (0.015)
Neighbourhood sports facilities (ref: no)	0.788* (0.610–1.019)	1.832** (0.966–3.475)	−0.039 (0.105)
Neighbourhood sports groups (ref: no)	1.089 (0.851–1.393)	0.659 (0.359–1.213)	0.054 (0.097)
Constant	1.167* (0.617–2.209)	0.003*** (0.001–0.016)	3.857*** (0.236)
Random part			
Variable (constant)	0.178***	1.345***	0.259***
Variable (residual)			1.150***
Number of individuals	5285	5285	5285
Number of neighbourhoods	405	405	405
AIC	6759.609	2482.315	16,295.660
Log likelihood	− 3361.8045	− 1223.1574	− 8128.8277

*Note. DV* dependent variable, *OR* odds ratio, *CI* confidence interval, *B* unstandardized coefficient, *SE* standard error, *AIC* Akaike information criterion. *p < .10, **p < .05, ***p < .01

We further examined whether a sedentary lifestyle mediates the relationship between neighbourhood population density and the risk of being overweight. The results of the analysis conducted with Model 4a, which is presented in Table 3, shows that the odds of being overweight decreased with higher amounts of time spent on physical exercise (OR = 0.903, 95% CI = 0.858–0.950). In addition, those owning a car were more likely to be overweight than those who did not own one (OR = 3.599, 95% CI = 2.833–4.571). The direct effect of neighbourhood population density on the risk of being overweight was significant (OR = 1.062, 95% CI = 1.019–1.108), even after controlling for the indirect effects of car ownership and time spent on exercise. However, results of the Sobel test and the multiple mediation test showed that both the amount of time spent on physical exercise and car ownership significantly mediated the relationship between neighbourhood population density and the risk of being overweight. Therefore, it can be inferred that these two indicators of a sedentary lifestyle partially mediated the relationship between neighbourhood population density and the risk of being overweight.

**Table 3:** Multilevel logistic regression estimates of odds ratios of being overweight (Model 4) and the mediating effect of lifestyle choice on being overweight (Model 5)

Variable	Model 4a	Model 4b
	OR (95% CI)	OR (95% CI)
Fixed part		
Independent variable		
Logarithm of neighbourhood population density	1.062*** (1.019–1.108)	1.027**(1.013–1.073)
Individual-level attributes		
Logarithm of time spent on physical exercise	0.903*** (0.858–0.950)	0.901***(0.856–0.949)
Motorized traveller (ref: non-motorized traveller)	3.599*** (2.833–4.571)	2.667***(2.048–3.472)
Male (ref: female)	0.921 (0.775–1.093)	0.924 (0.777–1.098)
Age	0.971*** (0.964–0.978)	0.970***(0.963–0.977)
Married (ref: single, divorced, or widowed)	1.220^+^ (1.000–1.488)	1.218*(0.998–1.487)
Education (ref: ≤ primary school)		
Junior high school	1.070 (0.911–1.256)	1.041 (0.886–1.223)
≥ Senior high school	1.001 (0.802–1.247)	0.881 (0.702–1.107)
Living in an urban area (ref: rural)	1.524*** (1.285–1.808)	1.504***(1.266–1.786)
Having meals ≤ three times a day (ref: meals > three times a day)	1.133 (0.933–1.376)	1.157 (0.952–1.406)
Smoker (ref: non-smoker)	0.741*** (0.626–0.877)	0.741***(0.626–0.878)
Drinking (ref: non-drinker)	0.871* (0.751–1.009)	0.867*(0.747–1.006)
Having medical insurance (ref: no)	0.943 (0.741–1.200)	0.941 (0.738–1.199)
Having a physical disease (ref: no)	1.751*** (1.538–1.993)	1.759***(1.545–2.004)
Logarithm of household income per capita	1.004 (0.978–1.030)	1.005 (0.979–1.031)
Neighbourhood-level attributes		
Logarithm of neighbourhood income per capita	0.993 (0.959–1.027)	0.991 (0.957–1.027)
Neighbourhood sports facilities (ref: no)	0.741** (0.582–0.944)	0.737**(0.578–0.941)
Neighbourhood sports groups (ref: no)	1.154 (0.908–1.466)	1.172 (0.920–1.494)
Cross-level interaction		
Logarithm of neighbourhood population density × Logarithm of time spent on physical exercise		1.001 (0.973–1.031)
Logarithm of neighbourhood population density × Motorized traveller (ref: non-motorized traveller)		1.476***(1.296–1.682)
Constant	2.016* (0.988–4.116)	2.641***(1.280–5.448)
Random part		
Variable (constant)	0.129***	0.133***
Number of individuals	5285	5285
Number of neighbourhoods	405	405
AIC	6627.554	6591.104
Log likelihood	− 3293.777	− 3273.552

*Note. OR* odds ratio; CI = confidence interva; AIC = Akaike information criterion. *p < .10, *p < .05, ***p < .01

The partial mediation effects [33] of car ownership and time spent on physical exercise indicated that other bio-psychosocial pathways (e.g. dietary habits and health knowledge) might have played a role in the relationship between neighbourhood population density and the risk of being overweight. Accordingly, the results of an analysis conducted using Model 4b revealed that respondents whose family owned a car in densely populated neighbourhoods were more likely to be overweight than those living in less dense neighbourhoods. However, there was no evidence to suggest that the amount of time spent on physical exercise moderated the linkage between population density and the odds of being overweight.

## Discussion

This study investigated the relationships between neighbourhood population density, a sedentary lifestyle, and the risk of being overweight in China which may help to improve people’s HRQoL. First, in contrast to previous studies that have focused on developed countries [22, 23], this study was conducted in a developing country. The findings of this study revealed that middle-aged and older people living in densely populated neighbourhoods have a higher risk of being overweight than those living in sparsely populated neighbourhoods in China. This relationship was found to be attributable in part to the former group’s greater likelihood of owning a car and spending less time on physical exercise. Population density was found to be negatively related to the risk of being overweight in Western developed countries. This may be the case because residents living in densely populated neighbourhoods are more likely to have low socioeconomic status, which renders them unable to afford cars [15, 17] and have more time for physical exercises such as walking [21, 24, 25]. However, this is not the case in China. One explanation for the detrimental effect of urbanization is related to the more frequent use of motorized private traffic and lower engagement in physical exercise among people living in densely populated urban areas in China (which is discussed in the following paragraphs). The other explanation is that rapid urbanization in China, which has resulted in cities becoming more heterogeneous, partitioned, and socially alienating, may make residents more stressed. Such a contention is consistent with the finding that rapid urbanization in China has increased citizens’ stress levels [34–42]. Given that increased stress is related to a higher risk of being overweight [30] such as by causing endocrine disorders [36, 42, 43], increased population density may increase the risk of being overweight by directly worsening residents’ anxiety. In addition, population density is positively associated with the density of fast-food restaurants in China; therefore, those living in densely populated neighbourhoods are more likely to consume high-calorie foods and have a higher BMI [44].

The results of our analysis showed that population density was positively associated with the odds of living a sedentary lifestyle (i.e. higher motorised private traffic and less physical exercise), which is inconsistent with those from developed countries [12–14, 17, 18, 21, 24]. Such a stark contrast between China and developed countries can be explained by cultural variations in the rural-urban divide and residential differentiation.

In China, high population density has made both public transport (e.g. overcrowded buses) and sidewalks inconvenient for middle-aged and older adults [27, 45, 46]. Thus, these individuals are more likely to drive a car and engage in less exercise in the form of walking, than those who live in less crowded areas. Furthermore, middle-aged and older adults typically do not prefer working out in a fitness centre [42, 43]; therefore, they may require open spaces and community-based sports facilities to engage in exercise. However, living in a densely populated neighbourhood makes this less likely and may cause older adults to stay indoors instead of exercising outside. In addition, in China, residents of a high socioeconomic status are more likely to live in densely populated neighbourhoods (which are normally located in inner-city areas), and are therefore, more likely to own private cars [47] and enjoy better facilities for physical exercise [26, 27, 48, 49].

In addition, consistent with previous studies, results from our analysis indicated that owning a car and spending less time on physical exercise might increase the risk of being overweight [17, 18, 26]. The emergence of an interaction effect also indicated that the effect of car ownership varied by neighbourhood population density. First, neighbourhood population density strengthened the effect of car ownership on respondents’ likelihood of being overweight, which may be accounted for by the reasons noted above. Interestingly, inconsistent with previous studies [2–5], we found that being a drinker and smoker increased the risk of being overweight in this study. The obesity paradox [50], which can be described as follows, is a possible explanation for this finding: Overweight people are more likely to be warned by doctors and are thus careful about their unhealthy lifestyles; this in turn may reduce the frequency of their drinking and smoking behaviours.

From a policy perspective, the present study findings suggest that, to reduce older residents’ risk of being overweight, government officials should pay attention to the rapid growth of population density. At the individual level, neighbourhood committees are advised to advertise more in order to inform middle-aged and older adults about the disadvantages of being overweight. At the neighbourhood and city level, environmental intervention measures (e.g. sports facilities, green space, more walkable sidewalks) should be implemented to encourage middle-aged and older people to actively engage in physical exercise. These could help to reduce the risk of being overweight and thus, improve individual’s HRQoL in their later life.

Despite the merits of this study, some limitations should be noted. First, we can neither infer the causality of the relationship between neighbourhood population density and the risk of being overweight, nor solve the selection bias that is implicit to the cross-sectional research design that was used in this study [51]. For example, in the case of China, wealthy people are more likely to live in densely populated neighbourhoods than poorer people, and the former group has a higher risk of being overweight than the latter group. Under such circumstances, there might be a bias in the estimated relationship between neighbourhood population density and the risk of being overweight. The problem of selection bias can be partially addressed by using advanced statistical techniques such as longitudinal data analysis and propensity score matching. Second, we demarcated the boundary of neighbourhoods based on the division of administrative areas, which may lead to a modifiable areal unit problem (MAUP) [52]. Third, the data used in the current study were collected in 2011; the neighbourhood environment as well people’s lifestyles may changed rapidly. For this reason, results from this study may not reflect the current situation in China. Fourth, the results of the regression analysis might have been influenced by self-report bias, as the time spent on physical exercise was reported by respondents themselves. Finally, although a larger dataset was available, only a subset of respondents was selected to answer questions about physical activities. Therefore, even though the CHARLS selected their respondents using random sampling method, there is a possibility of sampling bias in the present study. For example, some selected respondents, especially those who were overweight, might have refused to answer questions about their body weight, thereby excluding themselves from the study sample. If this is the case, the emergent relationship between population density and the risk of being overweight is likely to be an underestimate.

## Conclusions

This study enhances our understanding of the linkages between population density, a sedentary lifestyle, and the risk of being overweight, among Chinese middle-aged and older adults. Living in a densely populated neighbourhood may increase their likelihood of following a sedentary lifestyle, and in turn increase their risk of being overweight. Further research is needed to examine other bio-psychosocial pathways (e.g. dietary habits, stress levels) that could link population density to the odds of being overweight in the Chinese context.

## Data Availability

The datasets generated and/or analysed in the current study are available in the repository [http://charls.pku.edu.cn/zh-CN].

## Abbreviations

BMI: Body Mass Index
CHARLS: China Health and Retirement Longitudinal Study
PPS: Probability Proportional to Size
